# NRAMP1 Polymorphisms like Susceptibility Marker in Mexican Focus of Cutaneous Leishmaniasis

**DOI:** 10.1155/2016/7951285

**Published:** 2016-10-18

**Authors:** Mirsha Pamela Hernández-Rivera, Alicia Ramírez-Ramírez, Adelaido Chiñas-Pérez, Amalia Monroy-Ostria, Mario Eugenio Cancino-Díaz, Omar Hernández-Montes

**Affiliations:** ^1^Departamento de Inmunología, Escuela Nacional de Ciencias Biológicas, IPN, Carpio y Plan de Ayala, 11340 Mexico City, Mexico; ^2^Departamento de Infectología e Inmunología, Instituto Nacional de Perinatología, Montes Urales 800, 11000 Mexico City, Mexico; ^3^H. Ayuntamiento de Calakmul, Halaltun, 24640 Xpujil, CAM, Mexico

## Abstract

Cutaneous leishmaniasis (CL) is endemic in Campeche state, Mexico. Host and parasite factors are involved in the establishment and development of CL. Host factors include immune response and genetic background. NRAMP1 (Natural Resistance Associated Macrophage Protein 1) is important in innate immunity. Polymorphisms in* NRAMP1* have been associated with susceptibility or resistance to infectious and autoimmune diseases. To study the association of* NRAMP1* mutations with CL in patients from Calakmul, Campeche, samples from 115 CL patients and 69 samples of healthy people from the same area were evaluated. Five regions in* NRAMP1* were amplified and digested, looking for mutations in the promoter region (−524G/C), exon 3 (274C/T), exon 8 (823 C7T), and exon 15 (G/A) and deletion of 4 bp in the 3′UTR region. We found a statistical association between polymorphisms in 3′UTR region and exon 8 and CL [*χ* ^2^ = 13.26; *p* < 0.05; OR = 17.00; IC of 95% (2.24–128.99)]. Some patients who needed more than 40 doses of Glucantime® to heal injuries presented mutations in exons 3, 8, and 15. Multiple or ear lesions were not associated with* NRAMP1* polymorphism.

## 1. Introduction

Leishmaniasis is zoonotic disease, caused by different species of the genus* Leishmania*, protozoa transmitted to human and animal reservoirs by the bite of infected phlebotomine sandflies (genus* Lutzomyia*, in the new world) [1–3]. Leishmaniasis is endemic in the most tropical regions of the world; furthermore several factors like climate change, urbanization, deforestation, increased travel for tourist and work-related reasons, immigrations from endemic countries, and military operations can lead to an increase in risk of contracting the diseases in new areas [4]. In Mexico, four clinical forms of leishmaniasis are found, localized cutaneous leishmaniasis (LCL), diffuse cutaneous leishmaniasis (DCL), mucocutaneous leishmaniasis (ML) caused by* L. (L.) mexicana*, and visceral leishmaniasis (VL) [5, 6]; furthermore there are reports of* L. (V.) braziliensis* [5, 7] and* L. (L.) chagasi* [8]. The sylvatic region of the Yucatan Peninsula is an endemic focus of leishmaniasis; most of the recorded cases are LCL with few well limited ulcers located mainly on the ear, head, neck, and upper limb [9]. DCL is characterized by a large number of nodular skin lesions and MCL is a severe form with inflammations with progressive destruction of the mucocutaneous tissues. Both forms are rare in Mexico.

In murine models, the earliest phases and resistance to the infection with different species of* Mycobacterium* and other intracellular microorganisms like* Leishmania* are under control of NRAMP1 (Natural Resistance Associated Macrophage Protein 1) encoded by* NRAMP1* gene, previously called* Bcg*/*Lsh*/*Ity* (*Mycobacterium bovis* BCG,* Leishmania donovani*, and* Salmonella typhimurium*) which has 88% identity with the human formerly called* NRAMP1* [10, 11] and now called* SLC11A1* (solute carrier family 11 member 1 protein). NRAMP1 is expressed in the membrane fraction in late endosome and lysosomes in macrophages and polymorphonuclear leukocytes as a 90–100 KDa phosphoglycoprotein [12] and acts as a pH-dependent divalent cation carrier. This protein has been found in close association with natural resistance to infection with some intracellular pathogens, which plays a critical role in early innate macrophage responses to intracellular infection. Polymorphisms of human* SLC11A1* are associated with susceptibility to several inflammatory autoimmune disorders, as well as infectious diseases in African and Asian populations, including leprosy, tuberculosis, visceral leishmaniasis, and human immunodeficiency virus [12, 13]. Polymorphisms have also been suggested to modulate expression or alter the functional capacity of SLC11A1 to transport divalent cations, respectively [14]. In the case of leishmaniasis diseases, polymorphism of* SLC11A1* coupled with the expression of other genes has been associated with susceptibility to some forms of this disease in a specific Mexican endemic area [15] and specifically with CL in Brazil [16] and VL in Sudan [17] and Morocco [18].

Over five years of work in Campeche state, located in the Yucatan Peninsula, specifically in the municipality of Calakmul, endemic area for LCL, we have observed different behaviors of the clinical expression of CL and we found people living in this area who have never developed any form of CL to people who present with the disease more than once, all of them living under the same conditions. On the other hand, we have noticed that there is a variable response to Glucantime [19]. Some patients resolved CL with 15 to 20 doses and others required 40 to 100 doses to get the same effect. The host genetic background and immune response are important in the evolution of the infection. The purpose of this study was to evaluate the correlation between susceptibility or resistance to leishmaniasis with polymorphisms in NRAMP1 in patients with CL from Calakmul, Campeche.

The polymorphisms analyzed in this research were four single nucleotide polymorphisms (SNPs) identified in the promotor region (−524G/C), in exon 3 (274C/T), in exon 8 (823C/T), and in exon 15 (D543N) and one deletion in the 3′UTR region (1729 + 55del4). All of them related to resistance to intracellular microorganisms in humans.

## 2. Materials and Methods

### 2.1. Blood Samples

Blood samples were collected in the municipality of Calakmul, Campeche, from 115 patients with clinical and parasitologic diagnosis of CL. Patients, male or female over 10 years old, were included.* Leishmania* species was identified by PCR amplification.* Leishmania (L.) mexicana* was the causative agent in most of the cases, with few caused by* Leishmania (V.) braziliensis*. All the cases in the current study responded to antileishmanial therapy with Glucantime. Control group was defined as healthy individuals (*n* = 69), who never had the disease, are without scars or injuries related to CL, and are male or female over 10 years old. Each participant signed a letter of informed consent, and inclusion of young children consent was obtained from parents or guardians. The research was approved by the ethical committee of the Escuela Nacional de Ciencias Biológicas from Instituto Politécnico Nacional and with authorization from the municipality of Calakmul, Campeche, with the participation of its Public Health Department, in agreement with the International Ethic Guidelines for Biomedical Research Involving Human Subjects and the Norma Oficial Mexicana de Salud: NOM-003-SSA 2-1993, for sampling blood from human beings for diagnosis and therapeutics. The study was registered in the “Libro de Actas del Cabildo” with date May 24, 2002.

### 2.2. DNA Extraction

DNA was obtained from venous blood samples, and DNA extraction was performed using the Gentra Puregene Kit (QIAGEN, Germany).

### 2.3. NRAMP1 Genotyping

Polymerase Chain Reaction-Restriction Fragment Length Polymorphisms (PCR-RFLP) methods were used for genotyping four polymorphic regions (SNPs) of the NRAMP1 gene: substitution −524 G/C in the promoter region, substitution 274 C/T in exon 3, substitution 823 C/T in exon 8, amino acid substitution D543N G/A in exon 15, and one deletion 1729 + 55del4 (TGTG) in the 3′ untranslated region (UTR). The methodology used was as described by Saiki and colleagues [20], using sequence specific oligonucleotides which were previously described (Table 1) [21–24] and performed in a Gene Amp PCR Systems 9700 thermocycler (Applied Biosystems), using 0.20 mM of each dNTP, 50 pM of each primer, 1.5 mM MgCl_2_, 10 mM Tris pH 8.4, 50 mM KCl, 0.01% gelatin, and 1.25 U of* Taq* polymerase (Invitrogen Life Technologies, CA, USA) in a 50 *μ*L reaction. The PCR conditions for the five polymorphisms were the same: one cycle of 96°C for 5 min, 60°C for 60 sec, and 72°C for 60 sec, followed by 35 cycles of denaturation at 93°C during 45 sec, annealing at 60°C for 60 sec and extension at 72°C for 60 sec and one elongation step at 72°C for 10 minutes. The amplicons were analyzed by horizontal electrophoresis on 2% agarosa gels in TBE buffer at 110 V for 1 hour. The gels were stained with ethidium bromide (1 mg/*μ*L) and visualized using UV illumination system. RFLP were made using 10 *μ*L of amplicon in 20 *μ*L of reaction with one of the following restriction endonucleases:* Ava II*,* Fok I*,* Hinf I*,* Mnl I*, and* Nar I* for exon 15, 3′UTR, promoter region, exon 3, and exon 8 amplicons, respectively. According to the manufacturer's instructions (New England Biolabs and Invitrogen Life Technologies), restriction enzyme digestion products were resolved using a vertical electrophoresis, on 8% acrylamide gel, and were stained with ethidium bromide (1 mg/*μ*L) and visualized under UV light.

### 2.4. Statistical Analysis

Frequency in genotype differences between patients and control subjects was examined using the Chi-square test with two degrees of freedom and was considered statistically significant when *p* < 0.05. Further the previous analysis included odds ratio test, with a confidence interval of 95% to quantitatively assess the degree of association between polymorphisms in NRAMP1 gene and CL.

## 3. Results

### 3.1. NRAMP1 Polymorphisms

−524G/C; 274C/T; 823C/T; D543NG/A; and 1729 + 55del4 (TGTG) were evaluated by PCR-RFLP.  Table 2 shows details of the polymorphisms genotyped from 115 blood samples from patients with CL and 69 samples healthy controls, collected in the Calakmul municipality in Campeche state, Mexico.

823C/T polymorphism was significantly associated with CL infection (*p* < 0.05), a silent nucleotide substitution (C to T). The genotype C/T and the minor allele (T/T) were expressed in CL group while its expression is absent in the control group. This mutation was found related to the presence of the CL (*p* < 0.05, OR [CI = 95%] = 17.0) (Table 3). 3′UTR (1729 + 55del4) was significantly associated with infection of CL either; the mutated alleles del/del and TGTG+/del are present in CL patients and in minor proportion in the control group (*p* < 0.05, OR [CI = 95%] = 2.76).

In this study no association was detected with other polymorphisms analyzed when comparing patients with control group.

### 3.2. Polymorphism in NRAMP1 and Requirements of Glucantime Doses

The potential relationship between polymorphisms and requiring of higher dosages of Glucantime than the average dose of 25 ampules used in CL patients of the same region [25] was evaluated in this study. Association with polymorphism 274C/T in exon 3 of NRAMP1 was found. 11 of 13 samples presented the mutated alleles (Table 4), and two samples had the original allele. Association with polymorphisms 823C/T in exon 8 and D543NG/A in exon 15 of NRAMP1 was also found. Only 4 samples for exon 8 and 3 samples for exon 15 had mutated allele in homozygous way. It was not finding the heterozygous mutation in both cases. Analysis in the 3′UTR region shows no association. The correlation between the number of lesions and polymorphisms was also analyzed (results not shown) but no significant association was found.

## 4. Discussion

Several studies have established that* NRAMP1* is a gene coding for an important regulatory element in the tracks of macrophages activation and differentiation [24, 26].* NRAMP1* gene has been associated with susceptibility and resistance to diseases caused by intracellular pathogens, such as the protozoan* Leishmania*. In this study, we analyzed five polymorphic regions of* NRAMP1* in samples of patients with CL form and control people (without CL), four single nucleotide polymorphisms, and one deletion of four nucleotides in the 3′UTR region, in order to know their effect on the risk to develop CL after infection in an endemic area. Bellamy [25] has suggested a strong relationship between some polymorphisms in* NRAMP1* (5′(CA)n, INT4, D543N, and deletion in 3′UTR region) and the increased risk of acquiring tuberculosis in west area of Africa [26, 27]. However, other studies which involved patients from Korea, Japan, Brazil, and Denmark suggested no association between polymorphisms in* NRAMP1* and the risk of acquiring tuberculosis. Although it is the same disease, the results are different because the patients belong to different ethnic groups with different genetic background and clinical status [27]. Similar results were found with other diseases caused by intracellular pathogens in different populations. In* Leishmania* infection,* NRAMP1* polymorphisms could play an important role in the pathology of the disease, referring specifically to CL and VL susceptibility in an endemic area in Chiapas state, Mexico [15]. Therefore, we consider the study of the polymorphisms present in three exons in* NRAMP1* relevant if they could have high possibility of affecting the NRAMP1 protein function. In addition, the promoter region plays a decisive role in the expression of the protein, to change the receptor of transcription factors. And finally, a 4 bp deletion in an untranslated region plays a crucial role in posttranscriptional regulation gene expression. All of the above explain the observation made in the municipality of Calakmul, Campeche state, where some people acquire CL, while others, living in the same area, under similar conditions, never developed the disease. Also, we expected to find a correlation between the polymorphisms in* NRAMP1* and the tolerance presented in some patients, who need high doses of medication to heal CL.

In the present, no mutation in the promoter region (−524 G/C) (Table 3) was found, and 100% of the control people and patients with LCL presented the original allele (G/G). However, Donninger and colleagues [22] found that this polymorphism creates a site which binds a transcription factor and then the overexpression of NRAMP1, so this mutation has been associated with protection to infection due to hyperactivity of macrophages, but in this study, this mutation was absent.

Mutation in exon 3 274 (C/T) place was analyzed, and no significant differences in the genotype frequencies obtained were found between both groups, so mutation in exon 3 could not be associated with disease. Nevertheless Ortiz-Flores and colleagues [15] found an association between allele 274 C/C in exon 3 and susceptibility to developing CL in Mexican patients from two communities in Chiapas state, Mexico. The authors addressed that their finding differs from other studies realized in Sri Lanka and Brazil for the same diseases form, where no relation was found between this mutation and susceptibility to developing CL. The explanation for this could be that these two endemic areas in Mexico, Campeche and Chiapas states, are geographically widely separated, with different ethnic groups, and affected by different* Leishmania* species and probably by different variants of the same parasite species.

When exon 8 (823C/T) was analyzed, our results suggested that there is a significant association between this polymorphism and CL after applying Chi-square test (*χ* ^2^ = 13.26; *p* < 0.05) and the same with odds ratio test (OR = 17.00; CI = 95%), and we conclude that this mutation is a risk factor to develop CL when this mutation is present. This is because about 20% of patients with CL presented differences in this exon, unlike the control group, which merely happens in approximately 1.5% of the people of this group. Nucleotide changes in exon 3 and exon 8 in* NRAMP1* gene are considered silent mutations, since the change of a nucleotide or amino acid generates a change but may actually cause disease by altering regulatory elements affecting exon RNA splicing [28, 29]. In the regular edition of RNA, the primary transcript of a gene contains sequences encoding amino acids (exons) and long noncoding sequences (introns) that should not be present in the final mRNA. In each exon there are short nucleotide sequences called Exonic Splicing Enhancer (ESE), which function as enhancers of RNA splicing, indicating the editing complex, where there are the ends of exons [30, 31]. The binding of regulatory proteins of the edition to enhancer sequences in the primary transcript makes the spliceosome directed towards both ends of the intron and cleave the primary transcript before splicing the ends of exons. Synonymous changes in a nucleotide sequence of an exon could become invisible to the enhancer sequences to protein complex of the spliceosome (due to less affinity), so an entire exon could be excluded from the final RNA. Then the secondary structure of the mRNA is affected as the stability of mRNA [30, 32]. Additional studies suggested that decreased enzymatic activity due to reduced protein expression mediated by changes in the mRNA structures could explain the differences in the phenotype [32, 33]. Over 50 human diseases have been associated with synonymous mutations and, in a recent survey, of 21,429 polymorphisms associated with human disease, nonsynonymous and synonymous variations were determined to have a similar probability of disease association (1.46% and 1.26%, resp.) [34].

Although the polymorphisms present in exon 3 and exon 8 have the same effect on NRAMP1 protein, in our study only mutation in exon 8 showed an association with CL. These findings agree with the association found in Morocco with VL infections, while the same polymorphism seems not to be associated with CL infection caused by* Leishmania (V.) braziliensis* in Brazil [16].

In relation to polymorphism in exon 15 (D543N), the polymorphism consisting in the change of G for A, which generates a substitution of amino acid (aspartic acid/asparagine), affecting protein function, has been associated with susceptibility to tuberculosis in populations of West Africa and East Asia [27, 29]. When exon 15 polymorphism was analyzed in samples from CL patients, 65.2% presented the original allele and 34.8% presented both alleles. With samples from the control group, 75% presented original allele, 23% presented both alleles, and 1.4% presented mutated allele. After statistic analysis, no significant differences between groups were found, referring to genotype frequency.

Finally, polymorphisms in 3′UTR region of NRAMP1 gene were analyzed to find any association between CL susceptibility and deletion of four nucleotides in this region (−TGTG). 78.26% of samples from control people presented original allele and 21.73% were homozygous for the mutated allele. In the case of patients with CL 56.52% who had the original allele, 2.6% presented both alleles and 40.86% had mutated allele. After statistic analysis significant association was found between this polymorphism and CL after applying Chi-square test (*χ* ^2^ = 9.6; *p* < 0.05) and with odds ratio test (OR = 2.76; CI = 95%); then there is a risk factor to develop CL when this mutation is present. This is because about 43% patients with CL presented differences in the 3′UTR region, unlike the control group, which merely occurs in about 22%. The physiological effect of polymorphism in this region is not known exactly, but it is known that this region is considered gene regulatory elements [23] and its association with tuberculosis susceptibility in populations from Eastern Asia and Africa has been shown [35]. It is noteworthy that, unlike other regions in this study, patients with CL presented allele mutated in 40.86% and this allele was homozygous. In other regions, where we found a mutation, most were heterozygous. Some authors have mentioned that homozygous mutation has high relevance because the stability is greater [36] and draws attention to the high proportion of patients with mutated allele. Other authors have related the best transporter activity of NRAMP1 and its ability to generate an intraphagosomal ionic concentration (which allow survival and reproduction of microorganisms) and the presence of mutated allele and even heterozygote alleles [37]. This mutated allele also causes an appropriate level of Fe^+2^ that allows development of microorganisms because it has been suggested that affected NRAMP1 reduces iron release from macrophages. Then iron accumulates in the liver and spleen when erythrophagocytosis happens and when there are changes in level of iron and other metals, ROS- (Reactive Oxygen Species-) regulated signaling is altered, in the same way as inflammatory pathway activation, a very important antimicrobial mechanism [38].

In some studies, where macrophages with a mutation in NRAMP1 protein were infected with mycobacteria, the ability to process antigens was loose, affecting the CD4 lymphocytes activity and therefore the Th1 response, promoting a Th2 profile and the progression of tuberculosis [27, 35]. For the case of leishmaniasis diseases, a similar model is proposed, due to be an intracellular pathogen. Susceptibility to CL has been associated with several polymorphic genes, including* NRAMP1 (SLC11A1)* in Brazil. Another study realized in a Mexican endemic area (Chiapas state, Mexico) with the object related to polymorphism of different genes, including* NRAMP1*, with different forms of leishmaniasis diseases, showed that, for this population and endemic area, none of* SLC11A1* alleles analyzed (they chose seven polymorphisms of this gene) had no relation with susceptibility to CL, except for one genotype (C/C) in exon 3 (274C/T) which was associated with CL susceptibility [15]. This differs from results obtained with patients from Campeche state, where exon 3 polymorphisms in* NRAMP1* gene and CL susceptibility were not related. Maybe as mentioned before, the differences in the ethnic group,* Leishmania* species and geographical environment, may be the explanation for the results from Calakmul foci.

When the analysis was made with samples from patients with CL which needed higher doses of Glucantime than the mean of 25 ampules used by Vargas-Gonzalez [39], the association between polymorphisms (homo- and heterozygote mutation) in exon 3 caught our attention; 11 samples of 13 patients, which needed high doses of Glucantime, had a mutation in exon 3 and presented the allele (274C/T). We made a statistically important relationship between the presence of this allele in a patient and the need for high doses of Glucantime; the relative risk was found (OR = 3.94) (Table 4). Two samples had the original allele. Analysis of exon 8 and exon 15 shows association with risk of needing large doses of medication, but with OR values lower than those obtained in exon 3. When the UTR region for the same samples was realized, there was no association between high doses of treatment and polymorphisms in this region. Some samples showed an extra mutation in some other exons in addition to those existing in exon 3.

The correlation between the number of lesions and the amount of drug administered was also analyzed [results not shown] because patients who required high doses of Glucantime were the patients with many lesions, then 25 samples were analyzed and 22 presented, at least, one mutation in any of five regions of this study, and 3 patients had no mutation. Of 22 patients, 13 presented mutation in exon 3 and 2 patients presented mutation in exon 8; 3 patients had mutation in exon 15 and 2 presented mutation in 3′UTR region, unfortunately without statistical significance. There is clearly an alteration in NRAMP1 protein, affecting innate immunity, so the patient does not stop the growth of the pathogen and develops the disease again. Similarly, patients who had a single lesion were analyzed, and results showed that this group of patients at least had one mutation in some of the regions of this study, but no significant association was found.

## 5. Conclusions

In the case of leishmaniasis diseases, to date, there are no effective chemoprophylaxis measures as in malaria. Several groups are working to produce protective vaccines; others are looking for better treatment, less toxication, and more accessible. In Mexico there are no mechanisms for the prevention and control of leishmaniasis in short term. Unfortunately, new cases of CL arise in farming communities, who are looking for new lands to work in the jungle, where the life cycle of this parasite has prevailed. And as it was demonstrated in the population of this study, the probabilities of developing CL are higher if a person is susceptible due to mutation in exon 8 of* NRAMP1* or a deletion in 3′UTR.

Other polymorphisms of* NRAMP1* remain to be investigated in the same community and extend the study to other endemic areas in Mexico and what relation it has with CL expression. In the same way, genetic causes of the different response to drug must be investigated in depth, looking for the pharmacological activity protein genes.

## Figures and Tables

**Table 1:** Primers used in this study.

Polymorphisms	Primers
−524G/C	5′-AAC AAC TCT GAG AAG GGA CA-3′
	5′-TGT GCC CCA CAA CAC ATC TG-3′
274C/T	5′-TGC CAC CAT CCC TAT ACC CAG-3′
	5′-TCT CGA AAG TGT CCC ACT CAG -3′
823C/T	5′-CTT GTC CTG ACC AGG CTC CT-3′
	5′-CAT GGC TCC GAC TGA GTG AG-3′
D543N	5′-GCA TCT CCC CAA TTC ATG GT-3′ 5′-AAC TGT CCC ACT CTA TCC TG-3′
1729 + 55del4	

**Table 2:** Details of the polymorphisms genotyped and identified by PCR-RFLP in *NRAMP1*.

Polymorphisms	Localization	PCR product size	Restriction enzyme	Restriction enzyme products
−524G/C	Promoter region	585 bp	*Hinf I*	Allele 1 (G): 43 (2), 61, 62, 79, 297 bp Allele 2 (C): 43, 61, 79, 105, 297 bp G/C: 43, 61, 62, 79, 105, 297 bp

274C/T	Exon 3	216 bp	*Mnl I*	Allele 1 (C): 12, 37, 65, 102 bp Allele 2 (T): 12, 37, 167 bp C/T: 12, 37, 65, 102, 167 bp

823C/T	Exon 8	234 bp	*Nar I*	Allele 1 (C): 99, 135 bp Allele 2 (T): 234 bp C/T: 99, 135, 234 bp

D543N	Exon 15	240/244 bp	*Ava II*	Allele 1 (Asp): 39, 79, 122/126 bp Allele 2 (Asn): 39, 201/205 bp Asp/Asn: 39, 79, 122/126, 201/205 bp

1729 + 55del4	3′UTR	240/244 bp	*Fok I*	Allele 1 (+TGTG): 33, 211 bp Allele 2 (−TGTG): 240 +/−: 33, 211, 240

**Table 3:** Genotype frequency of the NRAMP1 polymorphisms.

Genotypes	CL (%)	Controls (%)	*χ* ^2^ value	OR (95% CI)
*Promoter region *−*524*				
Promoter region −524 G/G	115 (100)	69 (100)	Not statistically significant	
Promoter region −524 G/C	0	0		
Promoter region −524 C/C	0	0		

*Exon 3 274*				
Exon 3 274 C/C	48 (41.73)	35 (50.72)	Not statistically significant	
Exon 3 274 C/T	51 (44.34)	27 (39.13)		
Exon 3 274 T/T	16 (13.91)	7 (10.14)		

*Exon 8 823*				
Exon 8 823 C/C	92 (80)	68 (98.55)	13.26	17.00 (2.24–128.99)
Exon 8 823 C/T	18 (15.65)	1 (1.44)		
Exon 8 823 T/T	5 (4.34)	0		

*Exon 15 D543N*				
Exon 15 D543N G/G	75 (65.21)	52 (75.36)	Not statistically significant	
Exon 15 D543N G/A	40 (34.78)	16 (23.18)		
Exon 15 D543N A/A	0	1 (1.44)		

*3*′*UTR*				
3′UTR TGTG/TGTG	65 (56.52)	54 (78.26)	9.6	2.76 (1.40–5.46)
3′UTR TGTG/del	3 (2.6)	0		
3′UTR del/del	47 (40.86)	15 (21.73)		

*χ* ^2^ value = Chi-square value with *p* < 0.05 in the presence of the mutation and CL.

OR = odds ratio (95% confidence interval) presence of mutation is a risk for CL.

UTR = untranslated region. del = deletion.

**Table 4:** Genotype frequency of the *NRAMP1* polymorphisms in patients requiring high doses of Glucantime (HDG).

Genotypes	CL-HDG (%) *n* = 13	CL (%) *n* = 115	*χ* ^2^ value	OR (95% CI)
Exon 3 274 C/C	2 (12.5)	48 (41.73)	22.69	3.94 (0.83–18.59)
Exon 3 274 C/T	2 (12.5)	51 (44.34)		
Exon 3 274 T/T	9 (69.23)	16 (13.91)		

Exon 8 823 C/C	9 (69.23)	92 (80)	15.43	1.77 (0.50–6.28)
Exon 8 823 C/T	0	18 (15.65)		
Exon 8 823 T/T	4 (30.76)	5 (4.34)		

Exon 15 D543N G/G	10 (76.92)	75 (65.21)	31.75	0.56 (0.14–2.16)
Exon 15 D543N G/A	0	40 (34.78)		
Exon 15 D543N A/A	3 (23.07)	0		

3′UTR TGTG/TGTG	8 (61.53)	65 (56.52)	Not statistically significant	
3′UTR TGTG/del	0	3 (2.6)		
3′UTR del/del	5 (38.46)	47 (40.86)		

*χ* ^2^ value = Chi-square value with *p* < 0.05 in the presence of the mutation and requirement of high doses of Glucantime.

OR = odds ratio (95% confidence interval). UTR = untranslated region. del = deletion.
